# Primary Cilia in Pancreatic β- and α-Cells: Time to Revisit the Role of Insulin-Degrading Enzyme

**DOI:** 10.3389/fendo.2022.922825

**Published:** 2022-06-27

**Authors:** Marta Pablos, Elena Casanueva-Álvarez, Carlos M. González-Casimiro, Beatriz Merino, Germán Perdomo, Irene Cózar-Castellano

**Affiliations:** ^1^ Department of Biochemistry, Molecular Biology and Physiology, School of Medicine, University of Valladolid, Valladolid, Spain; ^2^ Unidad de Excelencia Instituto de Biología y Genética Molecular, University of Valladolid Consejo Superior de Investigaciones Científicas (CSIC), Valladolid, Spain; ^3^ Instituto de Salud Carlos III, Centro de Investigación Biomédica en Red de Diabetes y Enfermedades Metabólicas Asociadas (CIBERDEM), Madrid, Spain

**Keywords:** primary cilium, insulin-degrading enzyme, pancreas, β-cell, α-cell, insulin signaling, insulin, proliferation

## Abstract

The primary cilium is a narrow organelle located at the surface of the cell in contact with the extracellular environment. Once underappreciated, now is thought to efficiently sense external environmental cues and mediate cell-to-cell communication, because many receptors, ion channels, and signaling molecules are highly or differentially expressed in primary cilium. Rare genetic disorders that affect cilia integrity and function, such as Bardet-Biedl syndrome and Alström syndrome, have awoken interest in studying the biology of cilium. In this review, we discuss recent evidence suggesting emerging roles of primary cilium and cilia-mediated signaling pathways in the regulation of pancreatic β- and α-cell functions, and its implications in regulating glucose homeostasis.

## 1 Introduction

In 1676, Anton Van Leeuwenhoek was the first to discover the cilium and attribute it a motile function (1). However, the cilium was redefined as a critical organelle for development, homeostasis, regenerative processes, and regulation of signaling pathways in health and disease (2–4). Of note, studies in mammals have shown the relevance of cilia dysfunction in several pathologies. In humans, there are pathologies ranging from organ-specific disorders such as primary ciliary dyskinesia, hydrocephalus, polycystic liver and kidney disease, and retinal degeneration, to broad pleiotropic phenotypes such as Bardet-Biedl, Alström, and Meckel-Gruber syndromes (for a comprehensive review see ref. 5). Likewise, defects in cilia are associated with pathologies in rodents such as left-right asymmetry during mammals´ development (5), and kidney polycystic pathology (6). Consequently, the term “ciliopathy” refers to a spectrum of characteristic phenotypes with defects in ciliary structure and function (7).

### 1.1 Primary Cilium Structure

**Figure 1:**
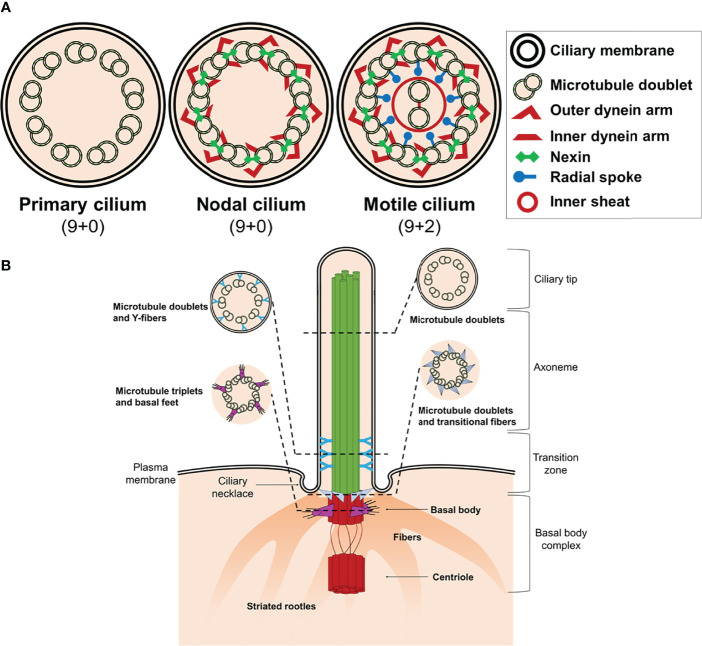
Structure of cilium. **(A)** Schematic representation of an axoneme cross section from a primary cilium, nodal cilium, and motile cilium. The axoneme cilium is composed of nine outer doublets of microtubules surrounding a central pair (9 + 2). The axoneme is ensheathed by a ciliary membrane. Inner and outer dynein arms, nexin, and radial spokes are responsible to link microtubules and form a cylindrical structure. **(B)** Schematic diagram of a typical non-motile primary cilium. The primary cilium is divided into the ciliary tip, the membrane bound axoneme extending from surface, the transition zone, and the basal body complex. The ciliary tip ends contain signaling molecules and can undergo morphological changes in response to signaling processes. The axoneme is the structural core of a cilium. The transition zone converts the triplet microtubular structure of the basal body into the axonemal doublet structure. The ciliary pocket (necklace) is an invagination of the plasma membrane at the root of cilium. The basal body complex comprises the basal body and its centriole. In most quiescent cells, the centrioles move to the apical plasma membrane and the basal body (mother centriole) functions as the microtubule-organizing centre to nucleate the axonemal microtubules. The centriole (daughter centriole) remains perpendicular to the basal body.

The majority of differentiated cells present a single cilium at the apical surface, while some cells accumulate bundles of cilia consisting of 200-300 individual organelles (8). Structurally, the cilium consists of a microtubule backbone (axoneme) ensheathed by a ciliary membrane that is continuous with the plasma membrane, which typically projects from the apical surface of cells (9). There are four main ciliary types referring to the axonemal organization of microtubules pairs: primary nonmotile or sensory cilium (9 + 0), motile (9 + 2), nodal (9 + 0), and non-motile cilium (9 + 2) (**Figure 1A** and **Table 1**). Nonetheless, the classic distinction between sensory and motile cilia seems to be very simplistic because there are motile cilia with sensory roles (8, 16–19).

**Table 1:** Main categories of cilia in humans (10–15).

Cilium type	Cells/Tissues	Functions
**Non-motile (9 + 0)**	Most quiescent cells of the body (e.g., tubular epithelia of the kidney, the bile duct, and pancreatic duct) Chondrocyte and osteocyte Photoreceptor cells	Sense environmental signals such as fluid flow and/or fluid composition Sense the amount of strain in bones Connect the inner and outer segments
**Motile (9 + 0)**	Embryonic node	Determination of left-right body asymmetry
**Non-motile (9 + 2)**	Inner ear (kinocilium, and stereocilia)	Mechanotransduction and auditory perception
**Motile (9 + 2)**	Respiratory epithelial cells, brain ependymal cells, and epithelial cells lining the fallopian tubes Spermatozoid flagellum	Transport extracellular fluid along the epithelial surface Motility in sperm

The basic structure of the primary cilium (9 + 0) is described elsewhere and it is out of the scope of this review (18, 20–22). Briefly, primary cilium consists of a ring of nine outer doublet microtubules (axoneme) which are devoid of dynein arms (**Figure 1A**). The framework of the doublet consists of a complete microtubule with 13 protofilaments (A-tubule) and an incomplete microtubule (B-tubule) with 10 protofilaments (21). The cilium also contains “matrix” proteins that are not tightly associated with either the membrane or the axoneme, including proteins required for assembly and maintenance of the organelle (23). The primary cilium structure can be considered an intricate network whose combination makes possible the integrity and function of the organelle. The literature describes almost 1,000 different proteins that exert some kind of function in the axoneme (24–26).

### 1.2 Ciliary Compartments

The cilium structure can be divided in ciliary and sub-ciliary compartments. The ciliary compartment consists in the axoneme and the ciliary membrane. The axoneme is a cylindrical structure of microtubules extended from the basal body, a specialized centriole structure, to the ciliary tip, which contains signaling molecules and can undergo morphological changes in response to signaling processes (**Figure 1B**). The ciliary membrane is continuous with the plasma membrane, but there are physiological differences between both. Thus, the ciliary membrane contains specific signaling molecules that are essential for the function of the cilium as antenna, including localization of ion channels and receptors at the base in an intra-membrane structure named ciliary necklace.

Microtubules in the axoneme are cylindrical polymers of α- and β-tubulin heterodimers. Post-translational modification of these tubulins (acetylation, methylation, tyrosination, phosphorylation, mono-glycylation, mono-glutamylation, poly-amination, poly-glycylation, and poly-glutamylation) may play a regulatory role on cilium mechanisms of action (27, 28). Most post-translational modifications occur on the external surface but, the Lys40 acetylation of α-tubulin (αK40) is located in the inner surface (or luminal side) of the microtubules (29, 30). Acetylation on αK40 most often stabilizes microtubules and it has been described to participate in the regulation of various signaling pathways by modulating the activity or the localization of plasma membrane proteins (28).

### 1.3 Sub-Ciliary Compartments

The structure and function of the sub-ciliary compartment is not fully understood. At the bottom of the cilium is found the basal body (commonly known as the mother centriole), a structure consisting of nine triplet microtubules arranged circumferentially (**Figure 1B**). The basal body maintains the transition zone between the axoneme and the cytoplasm of the cells and is attached to the membrane through the transitional fibers or alar sheets which go from the distal side of the triplets of the basal body to the cell membrane.

Another structure located to the sub-ciliary compartment is the striated rootlet, which is an array of periodically striated filamentous that radiate from the proximal end of the basal body to the cytoplasm of the cell (**Figure 1B**). The exact function of these rootlets is unknown, but it has been hypothesized to anchor the basal body/primary cilium complex with the cytoskeleton (31), participating as routes for the transport of proteins from the Golgi apparatus to the plasma membrane (32), and as structures that pull rapidly some primary cilia into the cell (33).

In addition, the protein turnover in primary cilium of pancreatic β-cells in mice is not uniform. Arrojo E Drigo and collaborators showed that the basal body contained high ^15^N levels, while the rest of the cilium was replaced by new components. These data suggest that long-lived structures are present in the basal body of β-cells leading to age mosaicism architecture within primary cilium (34).

### 1.4 Intraflagellar Transport

The elongation of the cilium requires targeting of specific proteins from the cytoplasm to the basal body area where pre-assembly of axonemal structures occurs, as well as the selective transport of proteins at the ciliary base out to the ciliary tip by the intraflagellar transport (IFT) system (35). When IFT particles are transferred from base to tip is referred to anterograde transport; and vice versa (from tip to base) is named as retrograde transport (reviewed in ref. 30).

Two IFT complexes play complementary roles in the transport of ciliary proteins. IFT complex A is required for retrograde transport, but seems not to be necessary for ciliary assembly. On the other hand, IFT complex B participates in the anterograde transport and it is essential for the assembly and maintenance of cilia. To avoid collision, IFT-A use the A-tubule, and IFT-B move along the B-tubule (36–39).

Additionally, two heterodimerized Kinesin-2 motor proteins and an accessory subunit [kinesin-associated protein (KAP)] catalyze anterograde transport of IFT complexes along microtubules to the ciliary tip, whereas retrograde transport of cargo proteins from the ciliary tip to the cytoplasm is catalyzed by cytoplasmic dynein 2 motor complex (36, 37).

## 2 Cilia Functions

The existence of four different kind of cilia indicates that the diversity of this organelle is intimately linked to different cellular functions.

### 2.1 Motile Functions

The motile function of 9 + 0 cilium at the embryonic node generate the nodal flow that is required for determine embryonic left-right asymmetry (5). On the other hand, the motile function of 9 + 2 cilium is required to move extracellular fluid. Thus, cilia of respiratory epithelial cells are responsible for mucociliary clearance (40). Likewise, the ependymal cilia facilitate ependymal flow (41), and the epithelial cilia in the female reproductive tract facilitate the movement of sperm to the site of fertilization (42).

### 2.2 Non-Motile Functions

There are non-motile functions such as those related with sensing environmental cues. In this case, cilia might act as antenna receiving signals due to the presence of receptors and ion channels in the ciliary membrane, which are transduced through intracellular signaling pathways. For example, monocilia from epithelial cells lining the mammalian kidney tubules have a mechanosensory role in sensing urine flow (43). Likewise, flow-sensing cilia of the periphery of the mammalian node seems to sense the leftward fluid flow generated by motile cilium within the node cavity (44). In addition, non-motile cilium are also important for the sensory apparatus of nose (45), eyes (46), and ears (47).

### 2.3 Primary Cilia and the Cell Cycle

On the other hand, cilia and the cell cycle seems to be coordinately regulated in many cells. Thus, the presence of the cilium is associated with the establishment of polarity and differentiation of the cells (G_0_/G_1_ phase). Conversely, the ciliated cells undergo a resorption of its cilium just before the beginning of cell division, when the cell leaves G_1_ phase and entry in S phase (48).

Dissecting the interplay between primary cilium and the cell cycle is an emerging area of research. So far, it is not well understood how extracellular mitogens (including serum stimulation), and genetic or pharmacological inhibition of ciliary regulatory proteins, contribute to ciliary dynamics and control of cell cycle progression (49, 50).

Studies using different cell lines have shown that serum starvation synchronize cell cultures in the G_0_/G_1_ phase leading to ciliary assembly. In contrast, serum-supplemented medium triggers two waves (fist between 1-2 h and second between 18-24h) of ciliary disassembly (51). Serum-mediated activation of Aurora A kinase (AURKA) induces ciliary disassembling by activating histone deacetylase 6 (HDAC6), which in turn regulates deacetylation of α-tubulin and cortactin, and ubiquitin-binding activity-mediated regulation of autophagy (51, 52).

The phospholipid mitogen lysophosphatidic acid (LPA), which is present at high concentrations in serum, is the major serum factor driving ciliary disassembly (53, 54). LPA notably triggers ciliary disassembling and subsequent cell cycle re-entry in serum-starved cells, in fact LPA is as effective as serum at inducing ciliary disassembling (55, 56).

Cell cycle-associated ciliary disassembly seems to occur *via* resorption, through depolymerization of the axoneme and incorporating its constituents into the cell body. However, ciliary disassembly in response to stress or pharmacological induction is mediated by whole cilium shedding, a process in which the ciliary membrane and axoneme are excised near the base and released from the cell (57–59).

## 3 Ciliogenesis

The primary cilium is formed from pre-existing centrioles, while multiciliated cells require new production of many centrioles (60). Once centrioles are formed in ciliary cells, they migrate to the cell surface, attach to the membrane and serve as basal bodies for ciliary elongation. During maturation, the centrioles acquire additional accessories such as transitional fibers and basal feet, which allow stabilization of the basal body/centriole (61, 62). The centriole was mostly known by its role in cell division, but its primary role maybe ciliogenesis, this idea is supported by the fact that cells can still divide without a centriole (48, 63).

Cilia are only assembled during the G_0_ (the period in which a cell remains in a quiescent and/or differentiated state). Conversely, entry into the cell cycle is preceded by ciliary resorption (48). Thus, the cilium has a dynamic structure, which is changing between growing and shrinking patterns and, is able to switch and quickly organize to other structures such as the mitotic spindle.

### 3.1 Extracellular and Intracellular Pathways of Ciliogenesis

Ciliogenesis occurs *via* two different processes named extracellular and intracellular pathways (64–66). In the extracellular pathway, prior to axonemal growth, the mother centriole directly docks with the plasma membrane from where the ciliary shaft is formed and grows towards the extracellular environment (67, 68). In the intracellular pathway, the mother centriole docks and fuses with the plasma membrane of an intracellular primary ciliary vesicle, where the cilium is assembled within the ciliary vesicle. Then, the elongated intracellular ciliary vesicle docks and fuses with the plasma membrane and the ciliary shaft is released into the extracellular space (67, 68).

Membrane trafficking regulators, such as the small GTPases Rab and Arl family, regulate the intracellular pathway (69, 70). Other trafficking regulators, such as components of the exocyst and TRAPPI/II complexes and SNARE membrane fusion proteins also play a role in intracellular ciliogenesis (71–73).

Finally, as we previously discussed, IFT is required for assembly and maintenance of cilium. Therefore, IFT is another process that regulates ciliogenesis. However, scarce information about sub-cellular localization of the IFT components have limited our understanding of the role of IFT during ciliogenesis.

## 4 Primary Cilium and Signal Transduction

An important function of the primary cilium is the regulation of key signaling pathways such as Hedgehog, Wingless, and insulin-like growth factor 1 (IGF-1)/insulin.

### 4.1 The Hedgehog Signaling Pathway

The Hedgehog (Hh) signaling pathway allows communication between cells during development. Although the pathway was discovered in *Drosophila melanogaster*, core components are conserved across flies and mammalian, but mechanisms of signal transduction have diverged (74–76). The Hh signal transduction in vertebrates is completely dependent on the primary cilium, and alterations in cilium structure, changes in protein activity or function, and changes in the location of proteins can influence Hh signaling. Cells lacking cilium are unable to induce the pathway in response to exogenous Hh ligands (77, 78).

**Figure 2:**
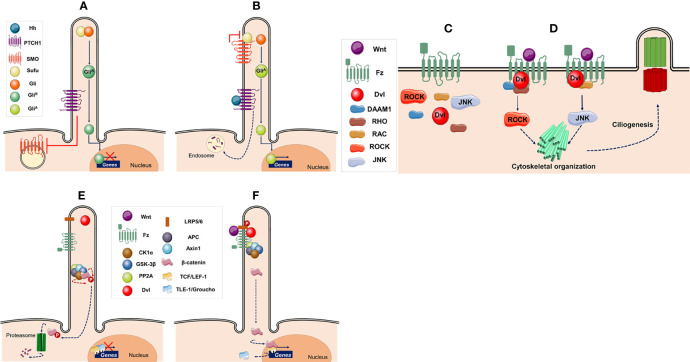
Ciliary signaling pathways. **(A–B)** Hedgehog signaling pathway **(A)** In the absence of hedgehog (Hh) ligand the intraflagellar transport machinery moves the transcription factor glioma-associated oncogene (GLI) and suppressor of fused (SuFu) to the ciliary tip. Patched-1 (PTCH1) located in the surface of the ciliary membrane inhibits Smoothened (SMO), which is located in the cytoplasm, keeping GLI in an inactive form (GLI^R^). The inactive transcription factor is transported back to the cell body and enters the nucleus where represses the expression of genes such as *CCND1*, *N-MYC*, G*LI1*, *GLI2*, and *PATCH1*. **(B)** Binding of ligand (Hh) to PTCH1 leads to the formation of oligomers, which are degraded in endosomes, reliving the repression of SMO, and causing its re-localization to the ciliary tip. SMO interacts with SuFu leading to the maturation of GLI into its active form (GLI^A^). GLI^A^ is transported back to the cell body and enters the nucleus where activates transcription of target genes. **(C, D**) Canonical Wnt signaling pathway. **(C)** Without Wnt ligands, β-catenin is ubiquitinated and degraded by the proteasome. The initial events of this pathway are regulated by a destruction complex composed of casein kinase 1α (CK1α), glycogen synthase kinase 3β (GSK-3β), protein phosphatase 2A (PP2A), adenomatous polyposis coli (APC), and Axin 1. In the absence of β-catenin, the nuclear T cell-specific transcription factor/lymphoid enhancer factor-1 (TCF/LEF-1)-responsive elements are associated with transcriptional suppressors, such as Groucho (Gro) and Transducin-like enhancer of split-1 (TLE-1), keeping the pathway inactive. **(D)** When Wnt ligands bind the Frizzled (Fz) family receptors and its coreceptor low density lipoprotein receptor-related protein-5/6 (LRP5/6), Fz recruits Disheveled (Dvl) to inactivate the β-catenin destruction complex. Thus, β-catenin accumulates in the cytoplasm and translocates to the nucleus where replaces Gro/TLE, and acts as a transcriptional co-activator with TCF/LEF-1, inducing transcription of Wnt target genes. **(E, F)** Planar cell polarity signaling pathway. **(E)** In the absence of Wnt ligands the pathway is inactive. **(F)** When a signal is received by the Fz receptor a complex of proteins, including Dvl, is recruited at the plasma membrane. Dvl activates RHO-associated coiled-coil forming kinase (ROCK) and c-Jun N-terminal kinase (JUNK) in parallel, resulting in cytoskeletal organization and regulation of ciliogenesis. This figure was created using Servier Medical Art (available at https://smart.servier.com/).

The functioning of the Hh pathway is based on the presence of the receptor Patched-1 (PTCH1) that is located in the surface of the ciliary membrane (79). In the absence of ligand, PTCH1 keeps the pathway off by inhibiting intracellular Smoothened (SMO; a Frizzled-Class-F G protein-coupled receptor). PTCH1 regulates the activity of SMO without direct interaction by changing the levels of cholesterol and/or cholesterol-derived molecules in the cell membrane (reviewed in refs. 74-76 (**Figure 2A**)). Without SMO activity, the suppressor of fused (SuFu) associates and represses the Glioma-associated oncogene (GLI; a zinc-finger transcription factor) at the ciliary tip, allowing that GLI protein undergoes proteasomal degradation leading to the formation of the transcriptional repressor (GLI^R^). The IFT machinery shuttles SuFu, GLI and GLI^R^ proteins from the ciliary tip to the cell body and vice versa. GLI^R^ enters the nucleus where represses the expression of genes under Hh control (**Figure 2A**) (80–82).

By contrast, binding of ligand (Hh) to PTCH1 leads to the formation of oligomers, which are moved out of the cilium and degraded in endosomes (83). This alleviates SMO repression causing its re-localization to the cilium, where switches off GLI processing by interacting with SuFu, leading to the activation of GLI (GLI^A^) (80–82). GLI^A^ is shuttle back to the nucleus allowing transcription of genes under Hh control. (**Figure 2B**) (79).

#### 4.1.1 Hh Signaling in Islets and β-Cells

Early reports by Thomas and collaborators demonstrated the presence of Hh, PTCH and SMO in rat and mouse islets of Langerhans (84). In addition, PTCH co-expressed with insulin in β-cells lending support to the notion that Hh signaling is not restricted to early pancreas development but also to functions of differentiated β-cells. In fact, Hh gain-of-function (ectopic expression) increases insulin production at the transcriptional level in INS1 and MIN6 β-cell lines. Conversely, loss of Hh signaling (cyclopamine) decreased endogenous insulin mRNA expression leading to diminished insulin content and secretion in INS1 cells (84).

The relevance of Hh signaling in β-cell function has been shown by Lau and collaborators (85). Thus, pancreatic epithelium elimination of SMO function (*Pdx1-Cre^early^;Smo^flox/null^* mice) resulted in a transient delay in β-cell development leading to a temporary reduction in β-cell numbers that were recovered after birth. However, adult knockout mice exhibited a mild insulin-dependent diabetes associated with glucose intolerance, reduced insulin secretion, and increased insulin sensitivity (85).

On the other hand, pancreatic epithelium overexpression of an activated version of GLI2 (*Pdx1-Cre;CLEG2* mice) failed to efficiently up-regulate the Hh pathway in the pancreas epithelium, suggesting the existence of a mechanism(s) that block inappropriate activation of the Hh pathway in epithelial cells (86). However, ablation of primary cilia in epithelial cells by specific deletion of *Kif3a*. resulted in strong activation of Hh signaling in mice harboring an activated version of GLI2 but not in SMO and PTCH1 mutant mice, indicating that primary cilia regulate Hh activity downstream of SMO in pancreas (86). Interestingly, the double transgenic mice (*Pdx1-Cre^ER^;CLEG2;Kif3a^f/f^*) showed lower expression of mature β-cell transcription factors, such as Pdx1, MafA, Ngn3, NeuronD1, and Nkx6.1. In addition, Hh target genes that are normally excluded from β-cells increased its expression, such as the precursor markers Hes1 and Sox9. Augmented Hh signaling resulted in impaired β-cell function and insulin secretion leading to glucose intolerance in double transgenic mice. Over time, the majority of double transgenic β-cells regained their differentiation state by downregulating GLI2 levels. Sustained Hh activity in the remainder of double transgenic β-cells resulted in neoplastic transformation of insulin cells into insulin-negative pancreatic tumors (87). These studies underscore the relevance of Hh signaling for maintaining β-cell function and identity.

Hh signaling has also been implicated in protecting β-cells from cytokine-induced cytotoxicity. Thus, Umeda and collaborators showed that proinflammatory cytokines increased Hh expression in rat islets and INS1E cells. Interestingly, Hh overexpression reduced cytokine-mediated apoptosis by decreasing nuclear factor-κB (NF-κB) promoter, whereas cyclopamine-mediated loss of Hh signaling increased cytokine-mediated apoptosis (88).

Similarly, Yalcinkaya and collaborators demonstrated that Hh signaling is involved in the mechanism by which high-density lipoprotein (HDL) protects β-cells from thapsigargin-induced endoplasmic reticulum stress and apoptosis. They showed that HDL involves the generation and mobilization of specific oxysterols and subsequent activation of SMO to elicit GLI^A^ nuclear translocation in INS1E cells (89).

The protective role of Hh signaling against cytokine- and –ER-induced apoptosis in β-cells await further confirmation *in vivo*, particularly in preclinical mouse models of type 2 diabetes (T2D), whereas both stimuli play important roles in the pathogenesis of the disease.

### 4.2 The Wnt Signaling Pathway

The Wingless-related integration site (Wnt) is an evolutionary conserved signaling pathway that regulates key features during development (90, 91).

Wnt signaling can be divided into two categories: canonical (or Wnt/β-catenin dependent) and non-canonical (or β-catenin-independent) (91, 92). The Wnt/β-catenin dependent pathway regulates stemness, cell differentiation and proliferation, whereas β-catenin-independent pathway regulates cytoskeleton, cell polarity, and cell movements (93, 94). Although for simplicity, Wnt signaling is often dichotomized in two arms, both pathways often overlap to organize complex cellular responses.

#### 4.2.1 Canonical Wnt Signaling Pathway

In the absence of Wnt ligands, β-catenin is ubiquitinated and degraded in the proteasome, keeping cytosolic β-catenin at low levels. Degradation of β-catenin is mediated by a “destruction complex” composed of casein kinase 1α (CK1α), glycogen synthase kinase 3β (GSK-3β), protein phosphatase 2A (PP2A), adenomatous polyposis coli (APC), and Axin 1. CK1α and GSK-3β sequentially phosphorylate the amino terminal region of β-catenin, resulting in β-catenin recognition by the F-box containing E3-ligase protein β-TrCP, an adaptor protein that forms a complex with the Skp1/Cullin machinery to attach ubiquitin to its binding partners, and subsequent ubiquitination and proteasomal degradation. In the absence of β-catenin, the nuclear T cell-specific transcription factor/lymphoid enhancer factor-1 (TCF/LEF-1)-responsive elements are associated with transcriptional suppressors, such as Groucho (Gro) and Transducin-like enhancer of split-1 (TLE-1), to keep the canonical Wnt pathway inactive (**Figure 2C**) (16, 95–98).

The outcome of canonical Wnt signaling is the expression of β-catenin target genes *via* β-catenin stabilization and subsequent nuclear translocation. Thus, extracellular Wnt binds to a complex of the Frizzled (Fz) receptor and the low-density lipoproteins receptor-related protein 5 (LRP5) or LRP6. Upon binding of Wnt, Fz receptor recruits the cytoplasmic phosphoprotein Disheveled (Dvl; A.K.A. Dsh) and GSK-3β is inactivated. In addition, Dvl recruitment by Fz leads to LRP5/6 phosphorylation, and Axin recruitment. These signaling events allow stabilization of β-catenin in the cytoplasm. Thus, β-catenin translocates to the nucleus, replaces Gro/TLE, and acts as a transcriptional co-activator with TCF/LEF-1, inducing transcription of Wnt target genes such as *cMYC*, *AXIN2* or *L1CAM* (**Figure 2D**) (8, 99–101).

#### 4.2.2 Non-Canonical Wnt Signaling Pathway

The non-canonical pathway refers to a group of Wnt-dependent signaling pathways that do not require LRP5/6 co-receptors and is β-catenin independent. The non-canonical pathway can be further classified into the planar cell polarity (PCP) and the Wnt/Ca^2+^ pathway (102). In this review, we only focus on the PCP pathway. The PCP pathway signals asymmetric cytoskeletal organization and coordinated polarization of cells within the plane of epithelial cells.

In the absence of Wnt ligands, components of the pathway are located in the cytoplasm (**Figure 2E**). When extracellular Wnt binds to Fz receptor, it recruits a complex of proteins at the plasma membrane that includes Dvl (103–105). Two independent and parallel pathways downstream Dvl led to the activation of the small GTPases RHO and RAC (**Figure 2F**) (106–110). The first pathway through the molecule Dishevelled associated activator of morphogenesis 1 (DAAM1) signals to RHO, which activates the RHO-associated coiled-coil forming kinase (ROCK) allowing regulation of cytoskeletal re-organization (109, 111, 112). In the second pathway, Dvl activates RAC, which in turn stimulates c-Jun N-terminal kinase (JNK) activity (**Figure 2F**), which also regulates cytoskeleton (110, 113).

#### 4.2.3 Wnt Signaling and Primary Cilium

Contrary to the connections between primary cilium and Hh signaling, the relationship between primary cilium and Wnt signaling is controversial (3, 114, 115) with the exception of the Wnt/PCP pathway, which affects cilia formation and functions *via* effects on cytoskeleton and basal body positioning (**Figure 2F**) (116, 117). There are reports showing that primary cilium disruption leads to upregulation of the pathway activity (118–121), and conversely, studies that refute any involvement of primary cilium in Wnt signaling (122, 123). Furthermore, two opposing models have been proposed regarding function of Wnt pathway in cilium formation: (i) a negative role of Wnt signaling in ciliogenesis (124, 125); and (ii) a direct role of the pathway in promotion of primary cilium (126–128). Even more puzzling, Bernatik and collaborators showed that neither activation nor deactivation of the canonical Wnt pathway affected the ciliogenesis (129). Thus, despite deep investigation, the function of the primary cilium in Wnt signaling remains unclear, particularly in pancreatic islets.

Corbit and collaborators have investigated the role of primary cilium in Wnt signaling generating three different transgenic mice in which *Kif3a*, *ITF88/Polaris*, and oral-facial-digital syndrome 1 (*Ofd1*) genes were knockdown resulting in impaired ciliogenesis (118). Genetic depletion of *Kif3a*, but not *ITF88/Polaris* or *Ofd1*, causes constitutive phosphorylation of Dvl and stabilization of β-catenin. Thus, primary cilium restricts the activity of the canonical Wnt pathway in mouse embryos, primary fibroblasts, and embryonic stem cells. Unciliated cells respond more robustly to Wnt stimulation than ciliated cells. Ciliary deficiency in pancreas also leads to activation of Wnt signaling (130). Thus, it appears that Wnt signaling is upregulated when cilia are absent, whereas cells with aberrant cilia structure downregulate the Wnt signaling pathway (131).

#### 4.2.4 Wnt Signaling in Islets and β-Cells

The impact of the canonical Wnt signaling pathway has been investigated using tissue-specific knockout mice in which β-catenin was ablated from β-cells. Thus, deletion of β-catenin early in development (*Pdx1-Cre,Catnb^lox/lox^* mice) resulted in reduced islets numbers, in agreement with the notion that the Wnt pathway is active in endocrine cells during development. However, in adult mice, where the pathway is not active in islets, glucose tolerance was normal in mice lacking β-catenin, suggesting that the Wnt pathway is not necessary for function or to maintain islet architecture later in life (132, 133).

On the other hand, deletion of β-catenin in the maturing β-cells (*RIP-Cre,Catnb^lox/lox^* mice) induced ~70% perinatal lethality and negatively impacts islets morphology and function as newborn mutant pancreas showed increased insulin content due to a defect in insulin release, and a reduction in total endocrine tissue. Nonetheless, the surviving mice showed mild glucose intolerance later in adulthood (134). These findings suggest that around the time of birth, where endogenous canonical Wnt signaling is activated in the endocrine pancreas, β-cells might be susceptible to loss of β-catenin signaling. However, this signaling might be dispensable in β-cells later in life.

The Wnt pathway is activated by numerous Wnt ligands generally divided into classical ligands (Wnt1, Wnt3a, and Wnt8) that activate the canonical pathway, and non-classical ligands (Wnt4, Wnt5, and Wnt11) that activate the non-canonical pathways. However, some Wnt ligands (e.g. Wnt3a) can activate both the classical and non-classical pathways underlying the cross talk between both pathways to organize complex cellular responses (135).

Krutzfeldt and collaborators analyzed activation of canonical Wnt signaling in adult pancreatic islets from wild-type (WT) and obese (*ob/ob*) mice *in vivo*. The canonical Wnt signaling did not occur in both WT and obese mice. Additionally, they identified the non-classical ligand Wnt4 as an abundant signaling molecule in adult mice islets that was upregulated in two different insulin-resistant mouse models [*ob/ob* and the lipodystrophic mice that lack adipose tissue (*aP2-SREBP-1c*)]. Furthermore, increased expression of Wnt4 inhibited canonical Wnt signaling in pancreatic islets and MIN6 cells (136, 137). These findings also highlight the complexity of the Wnt signaling in islets and β-cells since some non-classical Wnt ligands act as antagonists of the classical pathway.

### 4.3 Insulin Signaling Pathway

Many hormones and growth factors, such as insulin and IGF-1/2, act through receptor tyrosine kinases (RTK), including the insulin receptor (IR) and insulin-like growth factor receptors 1 and 2 (IGF-1/2R). In vertebrates, the three receptors (IR and IGF-1/2R) can bind with different affinities to insulin and IGF-1/2 (138). Of note, the three receptors can form a dimeric structure, which can be either a homodimer or a heterodimer, e.g., IRαβ/IGF-1αβR (139). The RTKs play a critical role in regulating cell proliferation, differentiation, survival, metabolism, migration, and cell-cycle control (140, 141). In the last decades, IR and IGF-1R have been linked to primary cilium and coordination of signaling events (142, 143).

Zhu and collaborators demonstrated that disruption of primary cilium assembly, by knockdown of IFT88 or the anterograde IFT motor subunit KIF3a in 3T3-L1 pre-adipocytes, reduced the ability of insulin to phosphorylate IGF-1R and AKT at the base of the primary cilium, leading to a decreased cellular expression of adipocyte transcription factors C/EBPα and PPARγ. Of note, a fraction of the cellular pool of IGF-R1 was detected in primary cilia of differentiated preadipocytes, and the receptors localized in the plasma membrane were less sensitive to insulin stimulation than those present in primary cilium (142). Likewise, Dalbay and collaborators demonstrated that differentiation of human mesenchymal stem cells into adipocytes required primary cilium elongation associated with recruitment of IGF-1βR onto the cilium (144). These studies demonstrate a link between insulin signaling through the primary cilium and cell differentiation.

**Figure 3:**
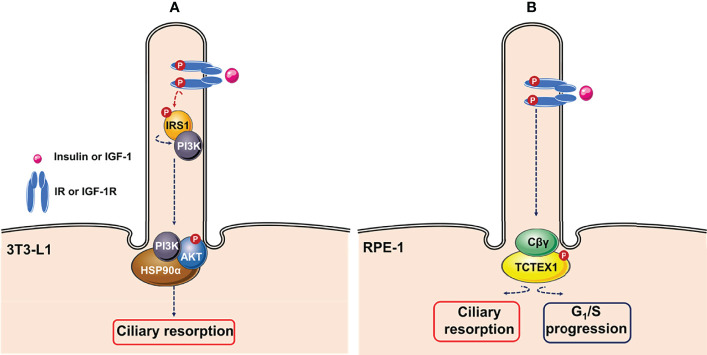
Ciliary insulin/IGF-1 signaling pathways. **(A)** Insulin stimulates resorption of cilia mouse in 3T3-L1 cells through activation of IGF-1R *via* recruitment of IRS1. Activated IRS1 is re-localized to the ciliary neck region, where heat shock protein Hsp90α might functions as a hub for activation of AKT. **(B)** Ciliary insulin growth factor-1 receptor (IGF-1R) activation induces ciliary resorption and G1/S progression *via* IGF-1-mediated recruitment of phosphorylated Tctex-type 1 (TCTEX-1) *via* non-canonical G-protein signaling (Cβγ) in RPE-1 cells. This figure was created using Servier Medical Art (available at https://smart.servier.com/).

Additionally, ciliary IGF-1R activation in 3T3-L1 cells also induces ciliary resorption. Wang and collaborators demonstrated that insulin-mediated activation of IGF-1R recruited and activated IRS-1, followed its re-localization to the ciliary neck region, where the heat shock protein Hsp90α might function as a hub for activation of AKT (**Figure 3A**) (145). Similarly, Yeh and collaborators showed that ciliary IGF-1R activation mediated recruitment of phosphorylated dynein light chain Tctex-type 1 (TCTEX-1) to the transition zone, leading to a mitogenic signaling cascade that accelerates ciliary resorption and G_1_/S progression in RPE-1 cells and cultured embryonic fibroblasts (**Figure 3B**) (146).

## 5 Primary Cilium Functions in Endocrine Pancreas

The endocrine pancreas is an important secretory gland in the regulation of glucose homeostasis due its capacity to secrete two glucoregulatory and antagonistic hormones, insulin and glucagon, in response to higher or lower blood glucose levels, respectively. The discovery of the islets of Langerhans, more than 150 years ago, made it possible to identify several endocrine cell types, most notably insulin-producing β-cells, glucagon-producing α-cells, and somatostatin-producing δ-cells, among other cellular types (147). For many years, insufficient insulin secretion was considered a culprit in the pathophysiology of T2D, but with the bihormonal hypothesis proposed by Unger and Orci this paradigm changed, stating that T2D resulted of the combined effect of hypoinsulinemia and hyperglucagonemia (148).

Primary cilium is found in endocrine pancreas (α-, β-, and δ-cells) (149–152), and exocrine pancreas (ductal cells of the Chinese Hamster, and centroacinar cells of bat) (153, 154), with the exception of acinar cells (130, 155). Several animal models of T2D have shown evidence of ciliary defects, suggesting a link between primary cilium and the pathophysiology of the disease. Thus, in the diabetes model of Goto-Kakizaki (GK) rat was observed a 3-fold reduction in primary cilia in β-cells, which was associated with missexpression of several ciliary/basal body genes (143). Similarly, several ciliary/basal body genes have been shown to be misregulated in pancreatic islets of the obesity and diabetes model *ob/ob* (156). A panel with mutations in genes related to cilia and their phenotypes in different animal models is shown in **Table 2**.

**Table 2:** Mutations in genes related to cilia and their phenotypes in several cellular and animal models.

Human gene	Model	Mutation	Pancreatic phenotype	Cell line phenotype	References
***BBS1***	Zebrafish	*Bbs1*-deficient	Increased β-cells mass and decreased α- and δ-cells cell types in early developmental stages	N/A	(157)
***BBS4***	Mouse Zebrafish Min6 cells	*Bbs4^-/-^* Downregulation of *Bbs4*	Mice: Islets size was not affected despite impaired glucose homeostasis and obesity onset. Insulin levels reported are either normal or increased Zebrafish: Increased β-cells mass and decreased α- and δ-cells cell types in early developmental stages	Loss of first phase insulin release. Unstimulated Min6 lacked both insulin receptor isoforms (IR-A or IR-B) in the cilium, after insulin stimulation IR-A, but not IR-B, was recruited to the cilium	(143, 157, 158)
***BBS5, BBS7, BBS9***	Min6 cells	Downregulation of *Bbs5, Bbs7*, and *Bbs9*	N/A	~2-fold increase in insulin secretion	(159)
***BBS12***	Mouse	*Bbs12^-/-^*	Islets size and plasma insulin levels were not affected despite enhanced *in vivo* insulin sensitivity	N/A	(160)
***AMLS1***	Mouse Zebrafish β-TC-6 cells	*Amls1^-/-^* Downregulation of *Amls1*	Mice: Pancreatic hyperplasia, partial degranulation of β-cells and islets cysts Zebrafish: Decreased β-cells production, hyperinsulinemia and impaired glucose-stimulated insulin secretion	Modest hypersecretory basal state in unstimulated cells, impaired glucose-stimulated insulin secretion and altered gene expression for signals downstream of glucose transport	(157, 161–163)
***AMLS1***	Mouse	*Alms1^L2131X/L2131X^*	Stunted cilia and loss of calcium signaling	N/A	(164)
***IFT88***	Mouse	*Ift88/Polaris^-/-^*	Loss of cilia in ductal and endocrine cells, cystogenesis, abnormal tubular structures, endocrine cells in duct, acinar cells apoptosis, endocrine islets normal except for increased β-cells clustering	N/A	(10, 130)
***KIF3A***	Mouse Min6 cells	*Kif3a^-/-^* Downregulation of *Kif3a*	Loss of cilia in ductal and endocrine cells, leading to acinar-to-ductal metaplasia, fibrosis, cyst formation, aberrant ductal cell morphology and lipomatosis	Decreased proliferation	(10, 165)
***HNF6***	Mouse	*Hnf6^-/-^*	Loss of ductal primary cilium, enlarged lumen and multiple cysts. Delayed Pdx1 expression and hypoplastic pancreas with retarded pancreatic specification of endodermal cells	N/A	(166–168)
***RFX3***	Mouse	*Rfx3^-/-^*	Reduced and stunned primary cilia. Reduced β-cells, α-cells, and δ-cells, increased pancreatic polypeptide-positive cells in perinatal stages. Adults showed small and disorganized islets, decreased insulin production, reduced glucose-stimulated insulin section, and impaired glucose tolerance	N/A	(155)
***LKB1/STK11***	Mouse	*Lkb1^-/-^*	Reduced β-cells, α-cells, and δ-cells number. Altered localization of cilia in β-cells and increased β-cells volume and insulin secretion *in vivo* with improved glucose tolerance	N/A	(169–172)
***IDE***	Mouse INS1E cells Mouse	*Ide^-/-^ in β-cells* Downregulation of *Ide* *Ide^-/-^ in α-cells*	B-IDE-KO: Impaired glucose-simulated insulin secretion. β-cell immaturity with constitutive insulin and pro-insulin secretion, decreased gene expression of *Ins2*, *Ucn3*, and *Pcsk1*, decreased GLUT2 plasma membrane levels A-IDE-KO: Impaired ciliogenesis, α-cells hyperplasia, and hypertrophy, impaired insulin inhibition of glucagon secretion, hyperglucagonemia and hyperinsulinemia, but normal glucose tolerance	Impaired GSIS N/A	(173, 174) (175)

### 5.1 Pancreatic Phenotypes Associated With Human Ciliopathies

Genetic studies of mutated genes associated with the primary cilium have begun to unveil a role for cilia in endocrine cells. Thus, ciliary dysfunction can cause inherited ciliopathies such as the Bardet-Biedl syndrome (BBS) and the Alström syndrome (AMLS).

#### 5.1.1 Bardet-Biedl Syndrome

BBS is characterized by multi-organ dysfunctions (obesity, retinal degeneration, polydactyly, renal and gonadal malformations, and learning disabilities) (176). BBS is a genetic disease caused by the combination of at least 19 genes. Among them, the most commonly mutated are *BBS1* and *BBS10* (177, 178). The link between ciliary dysfunction and BBS was established by the discovery of *BBS8*, which codifies for a protein localized to centrosomes and basal bodies (179). BBS8 is one of the eight proteins [BBS1, BBS2, BBS4, BBS5, BBS7, BBS8, BBS9, and BBS18 (A.K.A BBIP10)] that form a complex named the BBSome, which is a component of the basal body involved in trafficking vesicles to the primary cilium (180, 181).

Obesity-associated BBS is paradoxically by related to lower susceptibility to develop T2D early in life (2-6% prevalence in childhood), and higher insulin sensitivity and glucose usage (160, 182). In the last decades, several studies using cell lines and animal models have begun to unveil how primary cilium defects in BBS syndrome can explain the associated phenotypes. Thus, the *Bbs12^-/-^* mouse model (BBS12; a chaperone protein required for ciliogenesis during adipogenic differentiation of human mesenchymal cells) showed increased obesity, normal glucose tolerance and increased insulin sensitivity in the fat, recapitulating clinical features of BBS (160). Intriguingly, plasma insulin levels (in fasting and during an oral glucose tolerance test) and pancreatic islets size in *Bbs12^-/-^* were similar to control mice despite enhanced *in vivo* insulin sensitivity (160).

On the other hand, the *Bbs4 ^-/-^* mouse model exhibited impaired glucose homeostasis before the onset of obesity. As reported for *Bbs12* null mouse, serum insulin levels and pancreas histomorphometry remained unchanged (143), although the mutant strain reported by Eichers and collaborators exhibited higher plasma insulin levels (158). Disruption of basal body integrity in the *Bbs4* null mouse led to impaired first phase insulin secretion ex vivo and *in vivo* (143). Of note, insulin receptor is recruited to the cilium of insulin-stimulated β-cells. Ciliary integrity was required for activation of downstream targets of insulin signaling (PI3K/FoxO1), leading to a reduction of its targets Snap25 and Syntaxin1A (143).

Interestingly, downregulation of *Bbs5*, *Bbs7*, and *Bbs9* in the Min6 mouse insulinoma cell line LED to ~2-fold increase in insulin secretion (159). By contrast, downregulation of *Bbs4* resulted in a loss of first phase insulin release (143) in Min6 cells. Furthermore, unstimulated Min6 cells did not show the insulin receptor isoform A or B (IR-A or IR-B) in the cilium. Although IR-A, but not IR-B, was recruited to the cilium after insulin stimulation (143).

#### 5.1.2 Alström Syndrome

The ALMS is another ciliopathy resembling BBS that is characterized by obesity, insulin resistance, T2D, hypertriglyceridemia, hyperleptinemia, retinal dystrophy, hearing loss, short stature, pulmonary and renal dysfunctions, and cardiomyopathy (183–190). AMLS is characterized by a highly penetrant obesity, but unlike BBS displays higher childhood diabetes rates (75%). These discrepancies seem to be explained partly by severe insulin resistance observed in Alström patients, a condition that apparently is not present in BBS patients (184–186, 188, 189, 191, 192).

The progression from early impaired fasting glucose toward overt diabetes in Alström patients is mostly due to a progressive failure in β-cell insulin secretion without any further worsening of insulin resistance (183, 193, 194). AMLS is caused by loss-of-function mutations in the *AMLS1* gene, which is highly expressed in adult and fetal pancreatic islets (164, 195). Genetic ablation of *Amls1* in mice results in hyperplastic islets, partial degranulation of β-cells, and islets cysts (161, 162). AMLS1 is enriched at the basal body of primary cilium, and mutations result in stunted cilia, with cells showing a loss of calcium signaling (164, 196).

Interestingly, transient knockdown of ALMS1 (siRNA-*Alms1*) in the β-TC-6 mouse insulinoma cell line resulted in constitutive insulin secretion independently of glucose concentrations (163). This inappropriate insulin secretion was paralleled with altered expression of genes known to transmit signals downstream of glucose transport, suggesting a potential involvement of ALMS1 in glucose sensing (163).

### 5.2 Pancreatic Phenotypes Associated With Ciliary Dysfunction in Zebrafish

Using zebrafish models of BBS and ALMS syndromes, Lohd and collaborators showed that loss of *Bbs1* or *Bbs4* resulted in a significant increase of β-cells mass. Conversely, loss of *Alms1* gene led to a significant decrease in β-cells mass. Further investigations into the mechanisms underlying these phenotypes reveled increased susceptibility to cell death under prolonged exposure to high-glucose conditions in both disease models. Interestingly, although *Bbs1*-deficient β-cells were similarly susceptible to apoptosis, the overall maintenance of β-cells was likely due to compensatory increased proliferation. Of note, changes in β-cells mass were paralleled by a decrease in α- and δ-cell types during early developmental stages (157). These findings potentially implicate primary cilium as important regulator of β-cell plasticity, and if they would be translated into the clinical setting of BBS and ALMS, suggest that the differential susceptibility to suffer T2D in both syndromes may be related to the production and maintenance of β-cell mass (185, 186, 188, 189).

Consistent with hyperinsulinemia seen in ALMS patients (184, 188), loss of *Alms1* in β-cells of zebrafish also exhibited hyperinsulinemia and an impairment in glucose-stimulated insulin secretion (GSIS) (163). This observation was also confirmed in mouse β-TC-6 cells (163). These data suggest a role for ALMS1 in both β-cell proliferation and function.

### 5.3 Pancreatic Phenotypes Associated With Stunted or Absent Cilium

The *Ift88/Polaris* gene is an intraflagellar transport protein necessary for ciliary assembly whose expression is restricted to the pancreatic ducts and islets (2, 197, 198). In mice lacking expression of the *Ift88/Polaris* gene (the orpk mouse model) cilia were absent or shorter in the pancreas. Perturbation of excocrine and ductal cells cilia result in cystogenesis, formation of abnormal tubular structures, and appearance of endocrine cells in the duct. Unexpectedly, acinar cells undergo apoptosis, resulting in an overall loss of exocrine tissue and pancreas size. The endocrine islets cells, where IFT88/Polaris is highly expressed, looked normal except for increased β-cells clustering, due to the reduced mass of exocrine pancreas (10, 130).

The kinesin family member 3a (KIF3a) is required for the intraflagellar transport and cilia formation (199). It is highly expressed in non-diabetic human islets and in islets of obese non-diabetic mouse model when compared to their respective diabetic controls (199). Pancreas-specific ablation of *Kif3a* in mice (*Pdx1-Cre^early^; Kif3a^f^* ^/^ *^f^*) resulted in conditional loss of cilia in ductal and endocrine cells, leading to acinar-to-ductal metaplasia, fibrosis, cyst formation, aberrant ductal cell morphology, and lipomatosis. The use of different pancreas-specific *Cre* strains to knockdown *Kif3a* suggests that phenotypes seen in *Pdx1-Cre* mice might be caused by the absence of cilia in ductal cells. The pancreatic lesions in this mouse model resemble those found in patients with chronic pancreatitis of cystic fibrosis (10). Finally, the shRNA-mediated knockdown of *Kif3a* decreased proliferation of Min6 cells as well as dispersed primary mouse and human islets, providing direct functional evidence for the involvement of cilia in β-cells proliferation (165).

The hepatocyte nuclear factor 6 (HNF6) is expressed in the pancreatic progenitor cells and regulates the expression of pancreatic and duodenal homeobox1 (*Pdx1*) and differentiation to ductal cells (166, 167, 200). In pancreatic ducts, HNF6 controls primary cilium formation by regulating hepatocyte nuclear factor 1 homeobox B/transcription factor 2 (HNF1B/TFC2) and genes associated with cilium such as fibrocystin and cystin (167, 168, 201). Genetic ablation of *Hnf6* in mice resulted in an absence of primary cilium from pancreatic ducts and causing enlargement of the lumen and multiple cysts (167, 168). In addition, *Hnf6* null mice exhibited delayed *Pdx1* expression and a hypoplastic pancreas (166). This phenotype was not related to decreased proliferation or increased apoptosis, but from retarded pancreatic specification of endodermal cells (166).

Transcription factors belonging to the regulatory factor X (RFX) family are conserved along a wide range of species. In humans and mice have been identified five RFX factors (RFX1-5) (202). RFX3 is important for cilium formation by regulating expression of proteins necessary for cilium maintenance (155, 203). *Rfx3* gene is expressed in developing and mature pancreatic endocrine cells during embryogenesis and in adult mice (155). Endocrine cells of *Rfx3* null mice exhibited reduced number and severely stunned primary cilia. Consistently with the role of *Rfx3* in ciliogenesis, null mice showed uniquely altered cellular composition of the islets of Langerhans with fewer β-cells, α-cells, and δ-cells, whereas pancreatic polypeptide-positive cells were markedly increased in number during perinatal stages (155). The adult mice showed small and disorganized islets, decreased insulin production, reduced glucose-stimulated insulin section, and impaired glucose tolerance (155).

### 5.4 Pancreatic Phenotypes Associated With Defects in Cilium Organization

The liver kinase B1 (LKB1/STK11) is a tumor suppressor that acts *via* the activation of AMP-activated protein kinase (AMPK) (169, 204). In humans, *STK11* mutation is associated to Peutz-Jeghers syndrome, a condition characterized by high risk of pancreatic cancer and predisposition to gastrointestinal neoplasms (205). LKB1 is expressed in both - acinar and islets - cells during development and in early neonatal tissue, but in adults, it is expressed primarily in islets. Genetic depletion of LKB1 in mice (*Pdx1-Cre*; *Lkb1L/L*) resulted in pancreatitis, ductal cyst formation, abnormal cytoskeleton organization, defective acinar cell polarization, loss of tight junctions, and inactivated AMPK/MARK/SAD kinase family (170). *Lkb1*-null mice presented an exocrine phenotype that resembled the defect seen in the cilia of mice lacking *Kif3a* and *IFT88/Polaris* (10, 130). Additionally, *Lkb1^-/-^* mice showed an endocrine phenotype consisting in overall decrease in insulin-positive, glucagon–positive, and somatostatin-positive cells (170).

The primary cilia are generally found in the lateral surfaces of β-cells, which are normally arranged as rosettes around capillaries in islets. This organization of primary cilia relative to blood capillaries is important for β-cells insulin secretion as most exocytic processes happen in the vicinity of capillary beds (171). *Lkb1*-null β-cells presented altered localization of the cilia relative to islet capillaries, and they were not found in lateral surfaces of β-cells but localized to the cell surface opposite to the blood vessels (171, 172). Concordantly with this shift of cilia, *Lkb1*-deficient β-cells showed increased insulin secretion *in vivo* (169). Additionally, this change in cellular polarity resulted in hyperactivation of mTOR pathway, leading to a marked increase β-cell volume (65%) (169). Thus, LKB1 is not essential for ciliogenesis but instead influences cilia position regulating insulin secretion. On the other hand, LKB1 controls β-cell size independently of cilia polarization, because treatment of mice with rapamycin (an inhibitor or mTOR) restored normal β-cell size but did not reverse polarity defects of cilia (169). Finally, adult *Lkb1*-deficient mice exhibited faster glucose clearance in response to a bolus of glucose, which most likely is attributable to insulin hypersecretion (169, 170).

### 5.5 Pancreatic Phenotypes Associated With Loss of Insulin-Degrading Enzyme (IDE)

Insulin-degrading enzyme (IDE) is a ubiquitously expressed Zn^2+^-metalloendoprotease highly conserved and present in phylogenetically diverse organisms, ranging from viruses to humans. IDE is a multifunctional protein with proteolytic and non-proteolytic functions that was discovered more than 70 years ago by Mirsky and Broh–Kahn due its capacity to degrade insulin *in vitro*. In addition to this glucoregulatory hormone, IDE can also degrade glucagon and somatostatin (206, 207). Of note, genetic polymorphisms within or near the *Ide* locus have been linked to increased risk for T2D in different ethnicities, and mutations are associated with the development of T2D in the Goto-Kakizaki rat (206, 207). Also, genetic variations in and around the *Hhex/Ide* locus are associated with T2D incidence, decreased GSIS, and differential β-cell glucose sensitivity in response to an oral glucose challenge (207).

#### 5.5.1 Regulation of Insulin Secretion by IDE

As an initial approach to help elucidating the function of IDE on insulin metabolism *in vivo*, several laboratories developed mice with pancellular deletion of IDE (IDE-KO) and found age-dependent hyperinsulinemia and glucose intolerance (208, 209). To further decipher the role of IDE in pancreatic β-cells, Steneberg and collaborators found that GSIS was impaired by deletion of *Ide* (173). Furthermore, they showed that IDE levels were diminished by 40% in whole islets from T2D donors compared to controls (173), a finding later corroborated by Fernández–Díaz and colleagues *via* immunostaining (210). Of note, patients under oral hypoglycemic treatment showed decreased IDE levels in pancreatic β-cells, whereas insulin-treated patients showed increased IDE levels in β-cells relative to patients treated with oral hypoglycemic agents (210).

Fernández–Díaz and colleagues demonstrated a key role of IDE in regulating insulin secretion in mouse β-cells. shRNA-mediated silencing of *Ide* in the INS1E insulinoma cell line (INS1E-shRNA-IDE cells) resulted in decreased insulin secretion in response to glucose. Likewise, transient inhibition of IDE, with the specific inhibitor NTE-2, in rat and human islets resulted in abolition of GSIS (174). Furthermore, islets isolated from B-IDE-KO mice (*Ide*-deficient β-cells) showed a hypersecretory basal state in glucose-unstimulated islets accompanied by an impairment in GSIS (174).

#### 5.5.2 Primary Cilium and IDE in β-Cells

Interestingly, GSIS of B-IDE-KO isolated islets is similar to the observed in transient knockdown of *Alms1* and *Rfx3* in β-cells (155, 163, 164, 196, 203). Further findings support the notion that IDE may play a role on β-cell ciliogenesis. Thus, isolated islets from B-IDE-KO mice showed hallmarks of β-cell functional immaturity, such as constitutive insulin and pro-insulin secretion, decreased gene expression of *Ins2*, *Ucn3*, and *Pcsk1*, and decreased GLUT2 in plasma membrane (174). This phenotype also resembles the seen in the *Pdx1-Cre^ER^;CLEG2;Kif3a^f/f^* double transgenic mouse, which was devoid of primary cilium in β-cells and showed lower expression of mature β-cell transcription factors (87). Further research is warranted to demonstrate weather IDE has a role on β-cell ciliogenesis. To this end, B-IDE-KO mice and INS1E-shRNA-IDE cells would be important research tools to advance our knowledge on primary cilium in β-cells.

#### 5.5.3 Regulation of Glucagon Secretion by IDE

Fernández–Díaz and colleagues showed that IDE is differentially expressed in pancreatic islet cells, being expressed to substantially higher levels in pancreatic α-cells relative to β-cells and all other islet cell types (210). This finding suggests that it may be relevant to investigate the role of this protease in glucagon-producing cells to understand the molecular mechanisms underlying glucagon secretion. Merino and colleagues developed a mouse model in which *Ide* was knockout in α-cells (A-IDE-KO) that resulted in metabolic phenotypes consisting of hyperglucagonemia and hyperinsulinemia, but normal glucose tolerance. Importantly, *Ide*-null α-cells triggers hyperplasia, hypertrophy and impairment in cilia formation (175). The diversity of phenotypes seen in the A-IDE-KO mouse model indicates that IDE participates directly or indirectly in a number of cellular and physiological processes in α-cells. Deciphering the cause-effect relationship between IDE and each one of these phenotypes is an exciting opportunity but also a major challenge.

Closely paralleling the phenotype of constitutive insulin secretion produced by deletion of *Ide* from β-cells (174), α-cell specific-*Ide* deletion resulted in hyperglucagonemia (175). Two observations could explain, at least in part, this phenotype: (a) high-glucose and insulin levels failed to inhibit glucagon secretion in A-IDE-KO islets, and (b) the α-cell hyperplasia and hypertrophy.

The A-IDE-KO mice also exhibited hyperinsulinemia (175). This metabolic phenotype could be attributed to an exacerbated paracrine effect wherein excess glucagon release by α-cells stimulates the glucagon receptor on β-cells, leading to the activation of cAMP/PKA/EPAC pathway and thereby stimulating insulin secretion (211–215).

Unexpectedly, the A-IDE-KO mice showed α-cell hyperplasia (175). Based on published studies, it is plausible to hypothesize that this phenotype might be mediated by an interaction between IDE and the retinoblastoma protein (pRb), a tumor suppressor that inhibits cell-cycle progression at the G_1_/S transition when interacting with E2F transcription factors (216). IDE co-purifies with pRb on proteasomal preparations of breast cancer and hepatoma cells (217). Similarly, IDE has been shown to co-immunoprecipitate with the tumor suppressor PTEN, accelerating its degradation by SIRT4 in response to nutritional starvation stresses (218). Further research is warranted to decipher internal machineries that lead to α-cell division in the absence of IDE.

#### 5.5.4 Primary Cilium and IDE in α-Cells

**Figure 4:**
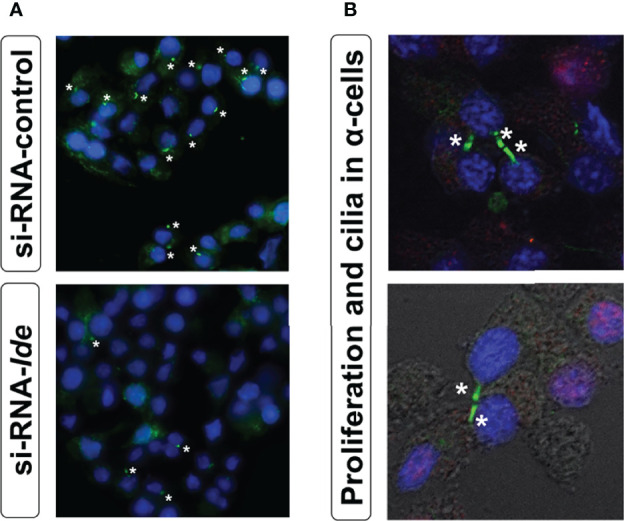
**(A)** Loss of IDE expression reduces ciliated α-cells number. Representative epifluorescence microscopy (40X zoom) images of cilia signal in siRNA-*Ide*- and control-treated α-cells. Acetylated α-tubulin (green) and DAPI (blue). Asterisks indicate the presence of cilia. **(B)** Primary cilium and proliferation in α-cells. Representative fluorescence microscopy images of cilia signal obtained with confocal microscopy (60X zoom) in non-permeabilized α-cells. As seen in the images, proliferation (BrdU staining) was associated with unciliated cells. Acetylated α-tubulin (green), DAPI (blue), and BrdU (red).

Genetic depletion of *Ide* in α-cells (αTC1.9-shRNA-IDE) resulted in cytoskeleton disarrangement and a significant reduction in the number of cilia (**Figure 4A**) (175). Classically, IDE has been viewed as a protease of insulin and glucagon, but this finding points towards non-proteolytic functions that could be regulating cytoskeleton integrity in pancreatic endocrine cells.

How non-proteolytic IDE function(s) may regulate ciliogenesis? IDE binds avidly to monomeric α-synuclein, leading to the formation of stable and irreversible complexes, thereby slowing the formation of higher-n aggregates of α-synuclein (173). In addition, IDE prevents α-synuclein aggregation in a non-proteolytical manner (219). Steneberg and collaborators first showed α-synuclein aggregation in IDE-KO β-cells. They assessed β-cell function in mice harboring pancellular deletion of *Ide*. They found impaired GSIS and reduced islet autophagic flux and microtubule content in IDE-KO islet cells. They also reported that IDE can form stable complexes with α-synuclein and that levels of α-synuclein were increased in islets from IDE-KO. Furthermore, IDE levels were inversely associated with increased levels of α-synuclein in islets of T2D patients (173).

Merino and collaborators showed that deletion of *Ide* in α-cells resulted in accumulation of oligomeric α-synuclein together with decreased levels of acetylated-α-tubulin (175). Acetylation of α-tubulin on Lys40 is a posttranslational modification associated with stable microtubules (220). The phenotype in α-cells is similar to that reported in β-cells from IDE-KO mice (173). Therefore, it is plausible to hypothesize that IDE loss of function causes α-synuclein oligomers formation that in turn reduces microtubule content and/or stabilization, which impairs assembly of axonema in primary cilia (221).

Hughes and collaborators demonstrated that primary cilia in β-cells mediate cross talk both within the islet and from islets to other metabolic tissues. β-cell specific depletion of cilia (*INS1-Cre/IFT88-Flox* mice) disrupts circulating hormone levels, impairs glucose homeostasis and fuel usage, and leads to the development of diabetes (222). In view of IDE-mediated regulation of primary cilium in α-cells, metabolic phenotypes of hyperglucagonemia and hyperinsulinemia seen in A-IDE-KO mice could be attributed to the absence of cilia leading to loss of cross regulation of β- and α-cells (175).

Similarly, the observed phenotype of α-cell hyperplasia and hypertrophy in A-IDE-KO mice could be related to loss of cilia. Merino and collaborators showed that in *Ide* depleted α-cells, lack of primary cilium was associated with increased proliferation (**Figure 4B**). Interestingly, proliferating α-cells exhibited a marked reduction in cilia abundance, an important hallmark of α-cell differentiation (175). The absence of cilia has been associated with increased proliferation in a variety of cell types, including pancreatic β-cells (223). The assembly and disassembly of primary cilium and lifecycle of centrosomes are tightly linked to cell division (165, 224–226). The absence of primary cilium could unleash the α-cells from a quiescent state, potentially triggering internal machineries that lead to α-cell division. Further research is warranted to unveil non-proteolytical roles of IDE in regulating cytoskeleton integrity, ciliogenesis and cilia-dependent cellular processes in pancreatic endocrine cells.

On the other hand, nonclassical chaperone activity mediated by synucleins is required for maintenance of continuous presynaptic SNARE-complex in neurons. α-synuclein directly binds to the SNARE-protein Vamp2 and promotes SNARE complex assembly (227). This evidence may explain why deletion of IDE in α-cells resulted in increased expression of genes coding for several members of the SNAREs protein complex, including *snap25*, *syntaxin1A* and *vamp2* (175). Because of the SNARE complex plays a key role in facilitating the fusion of glucagon granules to the plasma membrane, regulating cellular exocytosis, it is reasonable to hypothesize that these genes would be upregulated to meet the demand of continuous glucagon secretion (228). It is worthy to mention that SNARE proteins also play a role in intracellular ciliogenesis (71–73). Therefore, IDE might indirectly regulate ciliogenesis through its interaction with α-synuclein, which promotes SNAREs complex assembly.

**Figure 5:**
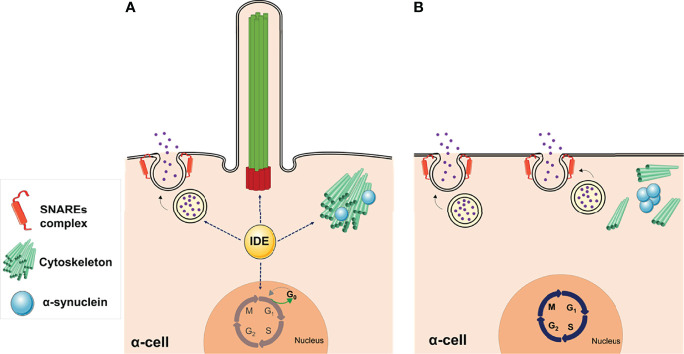
Non-proteolytic functions of IDE in α-cells. **(A)** The abundance of IDE in pancreatic α-cells is relevant for maintaining several cellular functions, such as glucagon secretion, cytoskeletal organization, and ciliogenesis, while cells are maintained in a quiescent state. **(B)** Deletion of IDE in mouse α-cells revealed multiple phenotypes, such as hyperglucagonemia, hyperplasia, and hypertrophy, suggesting different non-proteolytic functions of IDE in these cells. Lack of primary cilia along with increased proliferation, in *Ide* depleted α-cells, provide direct functional evidence for the involvement of cilia in α-cell proliferation. Likewise, loss of IDE causes α-synuclein aggregation, which might underlie the absence of cilia, cytoskeletal alterations, and augmented SNARE proteins of the secretory machinery. This figure was created using Servier Medical Art (available at https://smart.servier.com/).

In summary, B-IDE-KO and A-IDE-KO mice have uncovered novel non-proteolytical functions of IDE on ciliogenesis possibly through regulation of α-synuclein aggregation and/or SNAREs complex assembly. IDE deficiency leading to cytoskeleton disarrangements and ciliogenesis impairment in pancreatic α-cells, and most likely in β-cells, results in dysregulation of hormone secretion and cellular immaturity, which maybe triggering insulin and glucagon imbalance seen in diabetes (**Figure 5**).

## 6 Conclusions and Future Remarks

A growing body of evidence over the past decades have uncovered the importance of cilia in the maintenance of tissue homeostasis through paracrine and autocrine cellular communication. The notion that pancreatic cilia act as an antenna in which hormone receptors (such as insulin, glucagon, and somatostatin receptor) and regulatory proteins may change over environmental cues, enabling the cell to carry out specific functions in the maintenance of glucose homeostasis, opens new avenues for uncovering the pathophysiological processes underlying diabetes.

Current evidence suggests that primary cilium coordinate a variety of signaling pathways to control pancreas development, islets plasticity, and cell-type specific functions. While some signaling pathways are considered to be the bona fide ciliary pathways (e.g., Hh), others might be associated with cilia and probably act in cell-type specific manner, such as the insulin, glucagon, or somatostatin signaling. Although the prevailing idea considers the cilium as a sensory antenna, cilia also can participate as structural mediators of α- and δ-cells cross talk (222). This is especially relevant in the context of communication between islets cells that coordinate hormonal responses and maintain glucose homeostasis. Further research is warranted to discover intracellular crosstalk between cilium-dependent and cilium-independent signaling pathways in pancreatic islets.

Primary cilia are highly dynamic organelles whose configuration is tightly coupled to cellular proliferation and differentiation states. A major debate in pancreatic β-cell biology is focused on mechanisms (proliferation, progenitor differentiation, or trans-differentiation) governing plasticity of pancreatic islets cells in response to physiological conditions (e.g., pregnancy) and diseases (e.g., diabetes and obesity). In both T1D and T2D, reduced and/or inadequately β-cell mass leads to insufficient insulin secretion and hyperglycemia. Restoring β-cell mass and/or function is a major challenge in diabetes. Beyond this challenge, the study of β-cell ciliary biology is a promising possibility for improving β-cell regeneration and/or function that requires additional research.

For more than 70 years, emphasis has been placed on the study of the proteolytic functions of IDE. However, the biology of IDE has proven to be considerably more complex than expected. Tissue-specific knockout mouse models of IDE in endocrine pancreas (A-IDE-KO and B-IDE-KO mice) have demonstrated non-proteolytic functions of this enzyme. Particularly, its capacity to regulate the formation of α-synuclein aggregates strongly implicated in cytoskeletal integrity and vesicular trafficking. Thus, IDE is emerging as an important regulator of ciliogenesis in pancreatic cells, albeit molecular and cellular mechanisms remain obscure. IDE-mediated regulation of cilium seems to be of relevance to the processes of hormone secretion, inter-cellular communication, and islets plasticity to adapt environmental cues.

In conclusion, the extraordinary complexity of the primary cilium promises exciting future discovering such us the potential of cilia-related signaling molecules as therapeutic targets for new treatments fighting diabetes.

## Author Contributions

Conceptualization, MP, GP, and IC-C; writing—original draft preparation, MP, GP, and IC-C; writing—review and editing, MP, EC-Á, CG-C, BM, GP, and IC-C; supervision, IC-C and GP; funding acquisition, GP and IC-C. All authors have read and agreed to the published version of the manuscript. We apologize to those authors whose work has not been cited because of space limitations.

## Funding

The project leading to this Review has received funding from “La Caixa” Foundation, under agreement LCF/PR/PR18/51130007 to GP; Grants PID2019-110496RB-C21 and PID2019-110496RB-C22 funded by MCIN/AEI/10.13039/501100011033 to IC-C and GP, respectively; European Foundation for the Study of Diabetes Rising Star Fellowship to BM supported by EFSD-Novo Nordisk; This research was funded by the Programa Estratégico Instituto de Biología y Genética Molecular (IBGM), Junta de Castilla y León (CCVC8485). CG-C and EC-Á were supported by a fellowship from the Junta de Castilla y León and the European Social Fund (ORDER EDU/574/2018 and ORDER EDU/556/2019, respectively).

## Conflict of Interest

The authors declare that the research was conducted in the absence of any commercial or financial relationships that could be construed as a potential conflict of interest.

## Publisher’s Note

All claims expressed in this article are solely those of the authors and do not necessarily represent those of their affiliated organizations, or those of the publisher, the editors and the reviewers. Any product that may be evaluated in this article, or claim that may be made by its manufacturer, is not guaranteed or endorsed by the publisher.
